# Improving T-Cell Assays for the Diagnosis of Latent TB Infection: Potential of a Diagnostic Test Based on IP-10

**DOI:** 10.1371/journal.pone.0002858

**Published:** 2008-08-06

**Authors:** Morten Ruhwald, Janne Petersen, Kristian Kofoed, Hiroshi Nakaoka, Luis Eduardo Cuevas, Lovett Lawson, Stephen Bertil Squire, Jesper Eugen-Olsen, Pernille Ravn

**Affiliations:** 1 Department of Infectious Diseases 144, Copenhagen University, Hvidovre Hospital, Hvidovre, Denmark; 2 Clinical Research Centre 136, Copenhagen University, Hvidovre Hospital, Hvidovre, Denmark; 3 Liverpool School of Tropical Medicine, Liverpool, United Kingdom; 4 Zankli Medical Centre, Abuja, Nigeria; 5 Infectious Disease Unit, Department of Internal Medicine, Copenhagen University, Herlev Hospital, Herlev, Denmark; University College London, United Kingdom

## Abstract

**Background:**

There is a need for simple tools such as the *M.tuberculosis* specific IFN-γ release assays (IGRA) to improve diagnosis of *M.tuberculosis*-infection in children. The aim of the study was to evaluate the performance of an IP-10 and IL-2 based tests for the diagnosis of *M.tuberculosis*-infection in recently exposed children from Nigeria.

**Methodology and Principal Findings:**

Samples were obtained from 59 children at high risk of infection with *M.tuberculosis* (contacts of adults with smear and culture-positive tuberculosis) and 61 at low risk (contacts of smear-negative/culture-positive tuberculosis or community controls). IP-10 and IL-2 was measured in plasma after stimulation of whole-blood with *M.tuberculosis* specific antigens and mitogen. Previously developed criteria for positive IP-10 and IL-2 tests were used and the diagnostic performances of the IP-10 and IL-2 tests were compared with the Quantiferon In-Tube (QFT-IT) and the Tuberculin Skin Tests (TST). In response to *M.tuberculosis* specific antigens, the high-risk children expressed significantly higher levels of IP-10 (1358 pg/ml[IQR 278–2535 pg/ml]) and IL-2 (164 pg/ml[11–590 pg/ml]) than low risk groups 149 pg/ml(25–497 pg/ml), and 0 pg/ml(0–3 pg/ml), respectively. There was excellent agreement (>89%,*k*>0.80) between IP-10, IL-2 tests and QFT-IT, better than with TST (>74%,*k*>0.49). The IP-10 and IL-2 responses were strongly associated with *M.tuberculosis* exposure and with grade of infectiousness of the index cases (p<0.0001). IP-10, IL-2, and TST but not QFT-IT was associated with age of the child in the low risk groups (p<0.02).

**Conclusions/Significance:**

IP-10 is expressed in high levels and results of the IP-10 test were comparable to the QFT-IT. IL-2 was released in low amounts in response to the antigens and not in response to the mitogen therefore IL-2 seems a less useful marker. We have demonstrated that IP-10 and possibly IL-2 could be alternative or adjunct markers to IFN-γ in the diagnosis infection with *M.tuberculosis*.

## Introduction

Tuberculosis (TB) in children has long been neglected. The new WHO Stop TB Strategy, launched in 2006, aims at ensuring access to diagnosis and treatment of TB according to international standards, regardless of age[1]. Children recently exposed are at high risk of developing active disease. Preventive chemotherapy is effective, but is rarely used in high endemic regions despite WHO recommendations. This is due to the limited resources available, fear of emerging drug resistance and the lack of adequate tools for detection of infection in children. A major challenge for the wider implementation of targeted prophylaxis is the development of simple diagnostic tests for infection with *M.tuberculosis* (*Mtb*.).

With the identification of three *Mtb.* specific protein antigens (ESAT–6, CFP10, and TB 7.7), new diagnostic tests have emerged [2], [3]. These tests measure interferon gamma (IFN-γ) release after the stimulation of whole-blood (Quantiferon® –TB-Gold In Tube test (QFT-IT) (Cellestis Australia) or purified mononuclear cells (T-SPOT-TB®) (Oxford Immunotech) with the antigens and are known as Interferon Gamma Release Assays (IGRA). The antigens are contained within the RD1 and RD13 regions of the mycobacterial genome, which are absent from *M. bovis* Bacille Calmette Guerin (BCG), *M. avium* and most other non–tuberculosis mycobacteria [2], [4]

Although these tests are sensitive and specific for the detection of infection with *Mtb*. [3], [5]–[11], several reports suggest that their sensitivity may be lower in patients with active TB[3], [12], [13], that patients with HIV and severe immunosuppression may have an increased proportion of indeterminate results[12], [14]–[16], but there are only few studies including children.

Other biomarkers, alone or in combination, could enhance the diagnostic performance of IGRAs and we have previously reported that the IFN-γ-inducible protein 10 (IP-10) and (monocyte chemotactic protein-2 (MCP-2), and the interleukin(IL)-2 could be alternative or adjunct biomarkers to IFN-γ [17]

IFN-γ-inducible protein 10 has been designated the 10^th^ member of the CXC-chemokine family, CXCL10. It is predominantly produced by cells of the monocyte/macrophage lineage and is involved in trafficking monocytes and activated Th1 cells to inflamed foci through interaction with the CXCR3 chemokine receptor^18^, ^19^. High levels of IP-10 are found in the lymph nodes and tuberculous granulomas[20], in pleural effusions and broncoalveolar lavage [21], [22], in the serum of TB and TB-HIV co-infected patients experiencing the immune reconstitution syndrome[23], [24] and in PPD challenge of blood mononuclear and bronchoalveolar cells[24], [25]. IL-2 promotes T cell replication and is essential for cellular immunity and granuloma formation. *Mtb* antigen-dependent IL-2 production has been demonstrated in patients with active TB[26] and its serum concentrations are elevated in patients with active TB, returning to normal with treatment[27].

We have recently shown that QFT-IT has the potential to detect *Mtb* infection in recently exposed children from Nigeria in line with TST. We recently demonstrated a trend of increasing TST and QFT-IT responders with increasing numbers of bacilli in sputum of the index case [8] - a relationship which has been reported for the TST in other studies [28], [29]. The present study evaluated the potential performance of IP-10 and IL-2 as alternative or adjunct markers of *Mtb*. infection. In this setting we used the infectiousness of the index case to determinate the risk of infection of the child.

## Materials and Methods

### Objectives

The aim of the study was to evaluate the performance of previously defined IP-10 and IL-2 based tests for the diagnosis of tuberculosis-infection in recently exposed children from Nigeria.

### Participants

#### Study setup

Children were included as part of a study evaluating the risk of infection among children in contact with adults with culture positive pulmonary TB in Nigeria as described in [30].Children living in the households with adults who were diagnosed with culture positive TB were enrolled. Home visits were conducted between March and May 2005 >12 weeks after diagnosis of the index case. Eligible children were defined as any relative in the household <15 years of age who ate food prepared in the same cooking facilities as the index patient. During a home visit, a list of the children in the family was obtained, and <5 of these children were selected randomly to participate in the study. A thorough description of the recruitment and classification of the initial study population has been published [30] Culture was performed using BACTEC culture (Becton Dickinson, Sparks, MD, USA) by trained staff at Zankli TB Research Laboratory in Abuja. Results of the smear microscopy (bacillary load) of the adults were recorded according to the International Union Against Tuberculosis and Lung Diseases grading system as negative, scanty, +, ++ and +++ acid fast bacilli. Since all adults enrolled in the study had positive cultures, they were classified as having sputum smear-positive or sputum smear-negative TB Children were included >12 weeks after the index case was diagnosed and started on chemotherapy. We assumed that all infected would have developed *Mtb* specific immunity and we assumed that no further exposure took place although resistance testing was not done. Children included from a household of a sputum smear positive or negative adults were termed “SS+ contact” and “SS– contacts” respectively.

A group of children living in households with no known exposure to adults with active TB was included as community controls (CC). The HIV status of the adults was assessed as part of the routine investigations for TB (ImmunoComb HIVI and HIVII BiSpot kit (ORGENICS, Yavne, Israel)). Fifty (83%) of the adults were HIV1/2 tested and 28 (56%) were HIV-1 positive; none were HIV-2 positive. The HIV-status of the children was not known. The parents were interviewed using a questionnaire including medical history, degree of contact and clinical data including weight, height, and signs of active TB. Children with symptoms compatible with TB were referred to hospitals for further assessment and treatment. Chemoprophylaxis is not routinely offered in Nigeria, neither to children nor to HIV positive adults and was not given as part of this study. The incidence of TB in Nigeria is estimated to be 293/100.000[31], the prevalence of HIV-1 infection is estimated to be 6% of the adult population and 27% of the adults with TB are co-infected with HIV (>98% HIV-1) [32]. BCG-vaccination is routinely given at birth.

#### Blood sampling and biomarker measurement

From all children 3×1 ml of blood was drawn into vacutainer tubes coated with either saline (negative control tube), ESAT-6, CFP-10, and TB 7.7 peptides (*M. tuberculosis*–specific antigen tube), or the mitogen, phytohemaglutinin (PHA) (positive control tube). Tubes were transported to the laboratory in Abuja 2–6 hours after collection, and incubated overnight at 37°C. The tubes were then centrifuged, and the supernatant plasma was harvested and stored at −80°C. Later samples were transported to Hvidovre Hospital in Copenhagen on dry ice, and stored at −80°C. Samples were tested by ELISA for IFN-γ in 2006[8]. In 2007 supernatants were thawn and analysed by xMAP.

During the initial study[30] there was a shortage of mitogen tubes, and 79 of the 207 children enrolled were not tested with mitogen. For the present analysis, only samples where mitogen results were available were included. Thus for the present study, 128 of the 207 children initially screened with QFT-IT were evaluated for the presence of IP-10 and IL-2. There was no selection of who would be tested with mitogen as children were enrolled consecutively until stocks of mitogen tubes ran out. There were no significant age, sex, TST or QFT-IT differences between the children 128 included here and those not included.

### Description of procedures or investigations undertaken

#### Tuberculin skin test (TST)

Children were tested using the Mantoux method and 10 Units PPD (ChironVaccines Evans, Liverpool UK) equivalent to 5 IU tuberculin. TST readings were obtained by using the palpation method 48–72 hours later[8]. Induration ≥10 mm was used as cut off for positive result, and results using >5 mm and >15 mm were also calculated.

#### IFN-γ determination by Quantiferon

The QFT-IT test was performed according to the manufacturer's instructions as described previously[30]. Results are shown in pg/ml to facilitate comparisons with other biomarkers. One International Unit (IU) of IFN-γ corresponds to 50 pg/ml (NIBSC, Hertfordshire, UK). After subtracting the value from the negative control, the result was positive if, the antigen-dependent response was ≥0.35 IU(17.5 pg/ml), negative if the mitogen–induced response was ≥0.5 IU/ml (25 pg/ml) and the antigen-dependent response was <0.35 IU/ml(17.5 pg/ml), and indeterminate if both mitogen-induced and antigen-dependent responses were below cut-off or mitogen-induced response >8 IU/ml (400 pg/ml).

### IP-10, IL-2 and IFN-γ determination by xMAP and criteria for the diagnostic tests

IP-10, IL-2 and IFN-γ were measured by xMAP technology on the Luminex platform (Luminex Corporation), using Biosource reagents (Biosource Camarillo USA) acquired and analyzed with the STarStation v2.0 software (Applied Cytometry Systems) as previously described [17]. All measurements were performed in duplicate and blinded. The upper limits of quantification of cytokines using xMAP were 2,800 pg/ml for IP-10, 12,264 pg/ml for IL-2 and 27,200 pg/ml for IFN-γ. Cytokine concentrations above the limit of quantification were assigned the upper limit. The children were classified as positive, negative and indeterminate IP-10 test responders according to a test algorithm developed previously (Ruhwald et al, submitted). An IP-10 test was defined positive if: the antigen-dependent response was ≥455 pg/ml, negative if <455 pg/ml and the mitogen-induced response was ≥200 pg/ml, and indeterminate if both antigen-dependent and mitogen-induced responses were below cut off. IL-2 test algorithm was previously defined using 13 pg/ml as cut off [15]. An IL-2 test was defined positive if: the antigen-dependent response was ≥13 pg/ml and at least 125% of the nil value. (Details on the IP-10 and IL-2 test algorithms are provided in supporting information Methods S1).

### Ethics

The study was initiated and led by researchers from the School of Tropical Medicine and the University of Liverpool, both in Liverpool, UK in collaboration with Zankli Medical Centre in Abuja, Nigeria. Ethical approval for the study protocol, including permission to tests for IL-2 and IP-10, as described here, were obtained from the Institutional Review Board of these centres.

### Statistical methods

Data were analysed using SAS 9.1.3 (SAS institute). Variables with normal distributions were described using means and standard deviations and means were compared using Student's t-test. Median TST readings and biomarker responses were compared using non-parametic tests (Kruskal-Wallis, Wilcoxon rank sum test). ELISA and xMAP IFN-γ measurements were correlated using Spearman's rank test. The antigen-dependent and mitogen-specific biomarker production was measured by subtracting the concentration measured in the un-stimulated tube from the concentration measured in the antigen and mitogen tubes.

The concordance between TST, QFT-IT, IP-10, and IL-2 tests was assessed using Kappa statistics and McNemar's test. The Cochran-Armitage test for trend was used to correlate the prevalence of positive responders to the sputum smear grade of the index case and age. Fisher's exact test was used to compare differences in proportion of test-responders within groups. All tests were two sided and p values <0.05 were considered significant.

## Results

Samples from 128 children were tested for the presence of IP-10 and IL-2. Of these, 8 were excluded from analysis due to technical errors in the xMAP analysis (coefficient of variation >20% on more than 4 of the 9 set of measurements (n = 3) or lab handling errors (n = 5)). A total of 120 children had valid results and were included in the analysis. Fifty-nine (49%) children had been in contact with adults with SS+ TB, 38 (32%) with adults with SS− TB, and 23 (19%) were community controls, age and sex distribution is presented in table 1.

**Table 1:** Demographic data: number of children included, age, sex distribution and history of BCG vaccination stratified by exposure risk group.

	SS+	SS−	CC
N, (% of total)	59 (49)	38 (32)	23 (19)
Age (mean years (s.d.))	8.5 (3.5)	8.8 (3.9)	7.7 (3.7)
Under 5 y old, n(%)	8 (14)	7 (18)	5 (22)
Male sex (n (%))	23 (39)	16 (42)	12 (52)
BCG vaccinated n (%)	50 (85)	32 (84)	21 (91)

There were no significant differences between the groups.

### Comparing IFN-γ levels measured by xMAP or ELISA

The correlation between IFN-γ concentrations measured using QFT-IT ELISA and xMAP in samples stimulated with with *Mtb*-specific antigens and mitogen was very strong (r = 0.79 and 0.85, p<0.0001). There was no correlation in un-stimulated samples (r = 0.17, p = 0.07) as they were all very low. IFN-γ concentrations measured by xMAP were 3.5 fold (Inter quartile range 1.5–10) lower than ELISA concentrations (p<0.0001). Because of these low levels of IFN-γ measured by the xMAP we concluded that xMAP determination of IFN-γ would be too uncertain and we decided only to use IFN-γ measurements based on the QFT-IT ELISA for the subsequent analysis.

### Antigen-dependent IL-2, IP-10 and IFN-γ responses were associated with *Mtb* exposure

**Figure 1:**
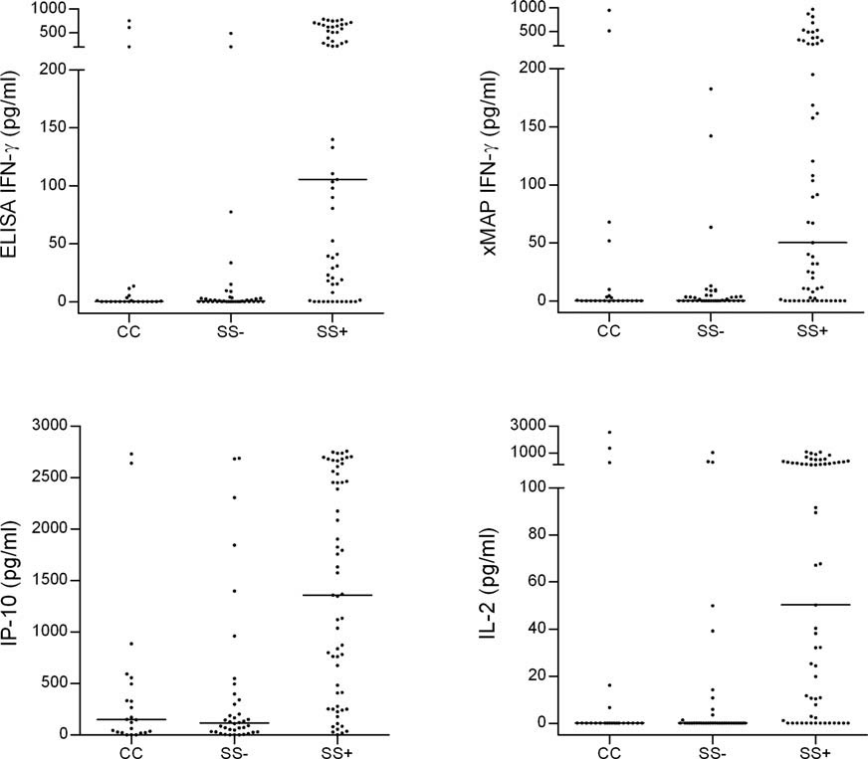
RD1 antigen dependent biomarker release in children with high and low risk of TB infection. Whole-blood from 59 contacts to a sputum smear-positive household adult (SS+), 38 contacts to a sputum smear-negative culture-positive adult (SS−), and 23 community controls (CC) was stimulated with RD1 antigens. Individual Interferon (IFN)-γ, IFN-γ-inducible protein (IP)-10, and Interleukin (IL)-2, responses after stimulation are shown (the values of the un-stimulated wells have been subtracted). Each dot indicate an individual response, horizontal lines indicate median.

Upon stimulation with *Mtb*-specific antigens, SS+ contacts released significantly higher concentrations of the three biomarkers than SS− contacts and CC (p<0.0001) did and there was no significant difference between SS− contacts and CC (p>0.4) (table 2 and figure 1). Median levels of IP-10 and IFN-γ after mitogen stimulation were 422 pg/ml (IQR 278–909 pg/ml) and 158 pg/ml (53–532 pg/ml) respectively, whereas the median level of mitogen-induced IL-2 was very low 5 pg/ml (0–254 pg/ml). There were no significant differences in the mitogen-induced levels of IP-10 and IFN-γ between the three groups (p>0.1), but the level of mitogen-induced IL-2 was higher in the SS+ group compared with the low risk groups (p<0.5). In un-stimulated samples, the concentrations of the three biomarkers were low and comparable between the groups (p>0.09).

**Table 2:** Production of IP-10, IL-2 and IFN-γ in un-stimulated, antigen-dependent, and mitogen-induced whole-blood from children exposed to sputum smear and culture positive adults (SS+), sputum smear negative but culture positive (SS−) adults, or community controls (CC).

		SS+ (n = 59)	SS− (n = 38)	CC (n = 23)
**IP-10**	Un-stimulated	106 (61–185)	143 (72–310)	126 (55–213)
	Antigen-dependent	1358 (278–2535)^a,b^	117 (32–341)	149 (25–497)
	Mitogen-induced	445 (250–708)	400 (231–983)	466 (105–934)
**IL-2**	Un-stimulated	1 (1–2)	1 (1–3)	1 (1–2)
	Antigen-dependent	164 (11–590)^a,b^	0 (0–5)	0 (0–3)
	Mitogen-induced	14 (5–21)^c,d^	3 (1–31)	5 (1–14)
**IFN-γ (ELISA)**	Un-stimulated	12 (9–29)^d^	24 (14–52)	17 (11–38)
	Antigen-dependent	106 (16–585)^a,b^	1 (0–3)	0 (0–6)
	Mitogen-induced	207 (63–449)	172 (50–289)	87 (17–433)
**IFN-γ (xMAP)**	Un-stimulated	1 (1–2)	1 (1–6)	1 (1–4)
	Antigen-dependent	54 (4–309)^a,b^	2 (0–7)	0 (0–8)
	Mitogen-induced	117 (44–227)	70 (17–181)	53 (13–127)

Data are shown as median concentrations in pg/ml,(25 and 75 percentile). Significant differences were found when comparing SS+ and CC (p<0.0001^a^ and p<0.05^d^), and when comparing SS+ and SS− (p<0.0001^b^ and p<0.03^c^).

### Identifying children at risk of LTBI using the QFT-IT, IP-10, IL-2 and TST

All four tests identified a significantly higher percentage of positive responders within the group of children exposed to sputum smear positive adults (table 3). Results obtained using QFT-IT was comparable to those obtained by the IL-2 and IP-10 tests. Among SS− contacts we found less QFT-IT responders compared to the IP-10 test (p<0.03, McNemar).

**Table 3:** Distribution of QFT-IT, TST, IP-10 test, and IL-2 test among children exposed to sputum smear positive adults (SS+), sputum smear negative, culture positive (SS−) adults, or community controls (CC).

	Test result	QFT-IT	IP-10 test	IL-2 test	TST 5 mm	TST 10 mm	TST 15 mm
		n/n total (%)	n/n total (%)	n/n total (%)	n/n total (%)	n/n total (%)	n/n total (%)
**SS+ contacts,**	Positive	42/59 (71)	42/59 (71)	44/59 (75)	36/53 (68)	24/53 (47)^a^	6/53 (11)^b^
	Negative	11/59 (19)	13/59 (22)	15/59 (25)	17/53 (32)	31/53 (53)	47/53 (89)
	Indeterminate	6/59 (10)	4/59 (7)	-	-	-	-
**SS− contacts, n/n total (%)**	Positive	3/38 (8)^a^	8/38 (21)	7/38 (18)	16/37 (43)^a^	7/37 (19)	1/37 (3)^b^
	Negative	28/38 (74)	21/38 (55)	31/38 (82)	21/37 (57)	30/37 (81)	36/37 (97)
	Indeterminate	7/38 (18)	9/38 (24)	-	-	-	-
**Controls, n/n total (%)**	Positive	3/23 (13)	6/23 (26)	4/23 (17)	6/21 (29)	2/21 (10)	0/21 (0)
	Negative	14/23 (61)	12/23 (52)	19/23 (83)	15/21 (71)	19/21 (90)	21/21 (100)
	Indeterminate	6/23 (26)	5/23 (22)	-	-	-	-

^a^Significantly different proportions of positive responders compared with the IP-10 test: p<0.03 ^b^Significantly different proportion of positive responders compared with the IP-10 test: p<0.009.

QFT-IT was indeterminate in 19 cases (16%), 14 were due to low mitogen responses and 5 due to a high nil response. There was no association with history of BCG vaccination and test results for any of the biomarkers or TST p>0.25). We were not able to demonstrate a difference in proportion of positive or indeterminate responders among any children <5 years in the SS + group (p>0.3). Diagnostic properties of the biomarkers independent of cut points are presented in Supporting Information Figure S1.

### Concordance between tests

The agreement between the QFT-IT and the IP-10 test results was very high (93/120, 78%, *k* = 0.64) and after excluding indeterminate responders the agreement between QFT-IT and IP-10 increased to 93/102, 89%, *k* = 0.80. (Table 4 and Table 5). The agreement between the QFT-IT test and the IL-2 test after excluding QFT-IT indeterminate tests 96% (97/101, *k* = 0.92). The agreement with the TST (>10 mm) and the biomarker tests were as follows: IP-10 test (69/93, 74%, *k* = 0.49), IL-2 test (88/111, 79%, *k* = 0.57), and the QFT-IT (70/93, 75%, *k* = 0.50). The concordance between the different tests is presented in supporting information Table S1 and Table S2.

**Table 4:** Head-to-head comparison of the IP-10 test and the QFT-IT for high risk (SS+) (kappa 0.62, agreement 83%).

		QFT-IT			
		Neg.	Pos.	Indet.	Σ
IP-10 test	Neg.	8	3	2	13
	Pos.	1	39	2	42
	Indet.	2	0	2	4
	Σ	11	42	2	59

**Table 5:** Head-to-head comparison of the IP-10 test and the QFT-IT for the low risk groups (SS− and CC) (kappa 0.50, agreement 72%).

		QFT-IT			
		Neg.	Pos.	Indet.	Σ
IP-10 test	Neg.	30	0	3	33
	Pos.	6	6	2	14
	Indet.	6	0	8	14
	Σ	42	6	13	61

### Association between *Mtb*. exposure and age with test results

**Figure 2:**
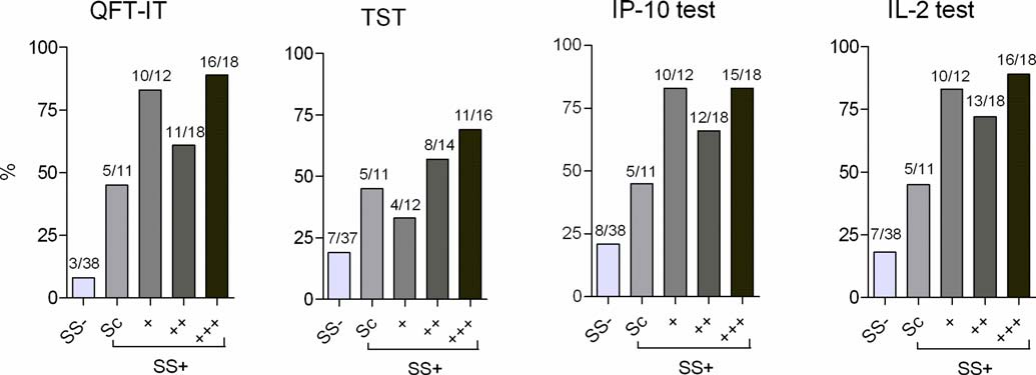
Association between exposure and test result. Proportions of children with positive, Quantiferon in tube test (QFT-IT), Tuberculin Skin Test cut off 10 mm (TST), IP-10 test, and IL-2 test results by household exposure are shown. SS−: Children exposed to adults with smear negative culture positive TB. Scanty (Sc), +, ++, +++ refer to the amount of acid fast bacteria seen in sputum smear microscopy. Indeterminate results are included. Numbers above bars indicate n positive/n total for each smear group. For all biomarkers and TST there was a significant trend for increasing test positivity rate by increasing smear grade among the TB contacts (p<0.0001).

**Figure 3:**
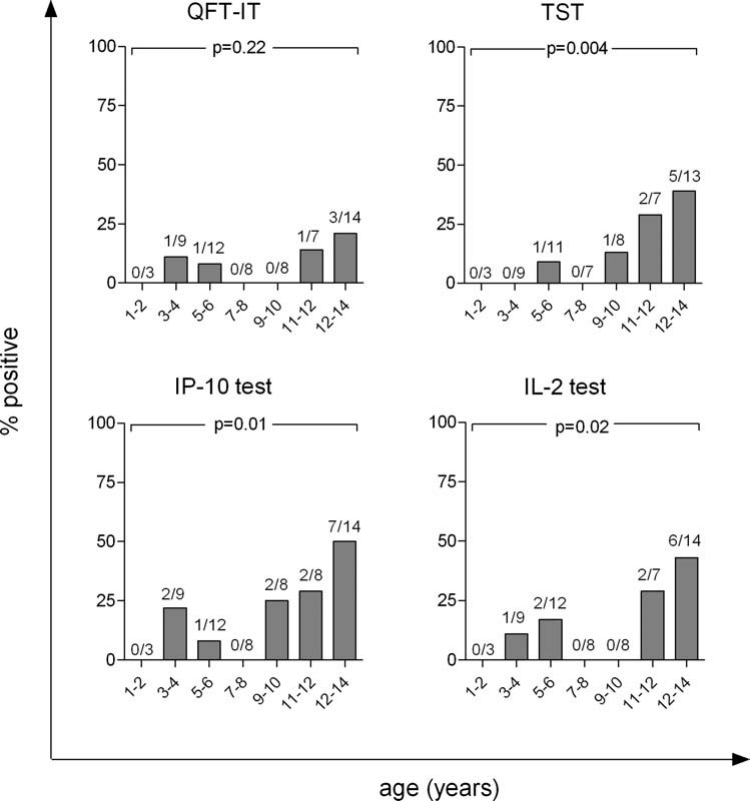
Association between age and test result. Proportions of community controls (CC) and sputum negative contacts(SS−) (n = 61) with positive, Quantiferon in tube test (QFT-IT), Tuberculin Skin Test (TST), IP-10 test, and IL-2 test result by age are shown. For IP-10 and QFT-IT, indeterminate results are included. For all tests except the QFT-IT there was a significant trend for increasing test positivity rate by age. Numbers above bars indicate the number of positive subjects per age group/n total for each group.

The proportion of children with positive tests stratified by the sputum smear grade of the index case was calculated (Figure 2). There was a significant trend for increasing test positivity rate by increasing smear grade among the TB contacts for all biomarkers (p<0.0001, Cochran-Armitage test for trend). The exclusion of indeterminate responders had no influence on the strength of the association. Using alternative cut points between 237 and 673 pg/ml for the IP-10 test did not change this strong association (data not shown),

It is well known that the risk of infection increases with age based on TST studies[28], [29] and we tested if there was a trend in test positivity rate with increasing age among the children. As all our results indicate that children of SS− adults appear to have a low risk of household exposure comparable to the CC, we pooled these two groups for the sub-analysis (n = 61). The SS− and CC did not differ significantly with respect to demographic data, antigen-dependent - and mitogen-specific biomarker levels, or test results. Figure 3 depicts the proportion of positive test responders by age groups. There was a significant age associated increase in rate of positive responders for the IP-10 test (p = 0.01), the IL-2 (p = 0.02) test, and the TST (10 mm) (p<0.004), but not for the QFT-IT (p = 0.22). When excluding indeterminate responders, this trend increased in strength and significance for the IP-10 test (p = 0.005), but there was still no trend observed for QFT-IT (p = 0.17). In contrast, there was no association between age and test positivity in the high risk group (SS+ contacts) for any of the biomarkers and TST (at 10 mm) (p>0.3)

### Combining biomarkers

We have previously demonstrated that a combined biomarker approach, classifying a responder positive if either the IP-10 test or the QFT-IT was positive, increased the sensitivity for diagnosing active tuberculosis (Ruhwald et al submitted). Using this combined biomarker approach, the rate of positive responders in SS+, SS− and CC groups were 45/59 (76%), 3/38 (21%) and 6/23 (26%) respectively, and the rate of indeterminate responders decreased to 2/59 (3%), 4/38 (11%) and 4/23 (17%) (table 6). Among SS− and SS+ the proportions of positive responders using the combined approach were significantly higher compared to QFT-IT alone (p<0.03 and p<0.05) and among SS− the rate of indeterminate results was significantly lower when compared to the IP-10 test (p<0.03). There were no significant increases in the rate of positive responders when combining the IL-2 test with the IP-10 test (p>0.3), or the QFT-IT test (p>0.08) (data not shown).

**Table 6:** Combining the results of a positive QFT-IT or IP-10 test results.

	Test result	Combined QFT-IT/IP-10 test n(%)
**SS+ contacts, n = 59**	Positive (either/or)	45 (76)^a^
	Negative (both)	12 (20)
	Indeterminate (both)	2 (3)
**SS− contacts, n = 38**	Positive (either/or)	8 (21)^a^
	Negative (both)	26 (68)
	Indeterminate (both)	4 (11)^b^
**Controls, n = 23**	Positive (either/or)	6 (26)
	Negative (both)	13 (57)
	Indeterminate (both)	4 (17)

Distribution of children exposed to sputum smear positive adults (SS+), sputum smear negative, culture positive (SS−) adults, or community controls (CC). ^a^Significantly different proportions of positive responders compared with the QFT-IT test: p<0.05 ^b^Significantly different proportion of indeterminate responders compared with the IP-10 test: p<0.03.

## Discussion

The study compares the performance of IP-10 and IL-2 with TST and QFT-IT for the diagnosis of *Mtb*. infection in children recently exposed to infectious cases of TB in a high endemic setting. The main findings are that a) IP-10 and IL-2 are expressed in high amounts in response to *Mtb.*specific antigens, b) The IP-10 test performs with excellent concordance and to the QFT-IT test and the agreement between the IP-10 and QFT-IT was stronger than with the TST, and c) The IP-10 and IL-2 responses were strongly associated with TB exposure as determined by exposure risk groups, by grade of infectiousness of the index cases, and by age of the child with low risk and d) combination of IFN-γ and IP-10 identified a more SS− contacts with no change in the control or SS+ group and there were fewer indeterminate results.

Childhood tuberculosis has long been neglected, but there is now focus on improving the diagnosis of active TB, and an increasing awareness of the use of prevention. WHO emphasizes that exposed children <5 years and HIV positive should be offered chemo-prophylaxis. However, blind prescription of Isoniazid may result in poor compliance and rapid development of resistance. Therefore targeted treatment of the infected subgroups would be a more rational approach than mass treatment. TST has been the only tool for the identification of infection in recently exposed children but it suffers from several operational and performance constrains such as low specificity, subjectivity in readout, dual visits, risk of boosting etc. A qualified alternative to the TST are the IGRAs using the RD1 antigens specific to *M.tb* [3], and we have recently shown that the QFT-IT was strongly associated with risk of recent exposure among children 1–14 years old in Nigeria[33]. Thus with tests based on RD1 peptides, targeted chemo-prophylaxis may become a realistic option even in high endemic countries.

As part of our search for improved and simple diagnostic measures we have compared the performance of IP-10 and IL-2 with QFT-IT in a diagnostic setting without a gold standard for infection, we used predefined cut offs and criteria for positive test results and exposure gradient as indicators for infection. We found that children 1–14 years old produced IP-10 and IL-2 in significant amounts after antigen-stimulation and we found a strong correlation between the biomarkers and risk of infection. Of the high risk children 71–75% were positive by IP-10 and IL-2 indicating a high sensitivity of a potential test based on IL-2 or IP-10.

The agreement between IGRA and TST varies between studies and ranges from excellent to poor[3]. There are several explanations for this discordance such as prevalence of TB, prior BCG, age at BCG vaccination, strain of BCG and batch of Tuberculin, age, co-morbidity etc. This matter has been intensively debated but there is consensus that the IGRAs are more specific for *Mtb.* infection, not influenced by prior BCG or exposure to NTM, and less affected by immuno-suppression[3]. Using the QFT-IT, as the comparator, we found excellent agreement between both the IP-10 and the IL-2 test. Although the performance of TST was good in this study the agreement with the TST and QFT-IT or IP-10 test was low, a finding which is consistent with other studies comparing TST and IGRA in high prevalence countries [34], [35]. In our previous study using exactly same IP-10 cut off and diagnostic algorithm, the positivity rate was 89% for patients with active TB and 3% for healthy unexposed students and we found 89% agreement between IP-10 test and QFT-IT test results. Together these studies strongly suggest that IP-10 responses are truly reflecting infection with *Mtb.* and not an unspecific chemokine release.

Nigeria has one of the highest TB rates in Africa. Therefore, community exposure is high and the prevalence of infection is anticipated to rise with age, hereby diluting the association with other risk factors [6], [30], [36]. Interestingly, our study found that among the low risk groups (the CC and SS−) there was a significant trend towards an increase in positive responders with increasing age for the IP-10, IL-2 and TST. This suggests that IP-10 and IL-2 actually did identify children infected by community exposure. We found no such trend in the high risk group which could be due to the fact that the majority of the high risk children were infected due to recent household exposure and not due community exposure increasing with age. The association between QFT-IT and increasing age was not significant, a result which remains to be explained or confirmed

Both QFT-IT and IP-10 test had high degrees of indeterminate responders (19/120, 16%, and 18/120, 15%, respectively). This could be caused by either poor sample handling or de-facto cellular anergy. A well described cause of inconclusive QFT-IT test results is HIV infection and low CD4 cell count[14], [16] and a limitation of this study was that the HIV status and CD4 cell count of the children were unknown and the number of families with known HIV status was too small for statistical analysis. The high degree of indeterminate responders underlines the importance of including a marker for cellular anergy.

The levels of PHA induced IFN-γ usually exceeds the levels of antigen induced IFN-γ. In the present study however, PHA responses were remarkably low and could have contributed to the high indeterminate rate. We do not think that this finding was due to low age of the children or to handling of the samples because there was no age-associated decrease in PHA and the responses to the specific antigens were comparable to our previous study[17].

IL-2 was not induced by phytohaemaglutinin stimulation a finding which has been reported by others[37]. Thus, it is not possible to develop an IL-2 based test algorithm with an option of indeterminate responses with the current set-up. Children with indeterminate IL-2 test results would therefore be classified as negative by default, which is a confounder when comparing results to the other tests. Still the IL-2 test had comparable performance to QFT-IT and IP-10.

In this and previous studies (Ruhwald et al submitted,[17]) the IP-10 test performed convincingly using whole blood stimulation and determination of IP-10 using xMAP technology. xMAP however, is not suitable for areas with limited resources. At present neither QFT-IT nor an IP-10 test using xMAP are affordable technologies for large scale screening of TB infection in low resource countries. There is still a need for improving the technology towards a simple, easy-to-use field friendly and cheap assay.

The present study had some limitation. First of all, the relative small sample size may have influenced the result of the analysis of association with age due to low numbers of children in each age category. However, the overall excellent agreement between the tests suggest that sample size was not too small for these comparisons.

The study was performed in Nigeria and the samples were transported to Denmark at a later stage which could have affected the quality. However, we have done stability studies showing that IP-10 and IL-2 are stable in plasma for at least 24 h at 20°C and are not degraded in plasma after >5 repeated freeze thaw cycles (unpublished).

Cut offs have been established using adult samples and it is possible that alternative cut offs should be used for testing in children. Our data does not suggest that age was a major problem as the positivity rate among children at high risk was >70% in all age groups and there was no age related difference in the mitogen-response. Only in the low risk groups we found an association with age which could be explained by increasing *Mtb* exposure with age in a high endemic region.

In conclusion, we have shown that tests based on determination of IP-10 and IL-2 perform with excellent concordance to the QFT-IT test. IP-10 and IL-2 responses are strongly associated with TB exposure as determined by exposure risk groups and by grade of infectiousness of the index cases. In addition IP-10 response was associated with increasing age in the child with low risk of infection.

We have provided data suggesting that IP-10 may serve as an alternative and/or adjunct marker to IFN-γ in the diagnosis of active as well as latent TB infection and that IP-10 may improve the overall diagnostic performance of the RD1 specific in vitro tests. IL-2 holds similar properties but is released in low amounts and there is no useful marker for anergy. As IP-10 is expressed in high amounts, it holds promise for the development of a new generation TB tests such as the lateral flow dipstick. Simpler and cheap tests are needed to facilitate the management of TB by increasing test accessibility and targeted preventive treatment especially of infected HIV+ve patients and in children.

## Supporting Information

Figure S1 Biomarker and Tuberculin Skin Test performance. Antigen-dependent Interferon (IFN)-γ, IFN-γ-inducible protein (IP)-10, and Interleukin (IL)-2, and the Tuberculin Skin Test (TST) readings (in mm) were analysed by receiver operating characteristic (ROC) curves. Result of ROC-curve analysis of the diagnostic potential of antigen-dependent IFN-γ, IL-2, and IP-10 and TST is shown. Community controls (CC) were used when estimating specificity and contacts of sputum positive adults (SS+) were used when estimating sensitivity. The cut offs with the highest proportional diagnostic strength (or maximum Youdens Index, sensitivity+specificity-1) were 635 pg/ml for IP-10, YI = 0.56, sensitivity 69%, Specificity 87%); 13 pg/ml for IL-2, YI = 0.57, sensitivity 75%, specificity 83%; 15 pg/ml for IFN-γ, YI = 0.65, sensitivity 78%, Specificity 87%; and 6 mm for the TST, YI = 0.44, sensitivity 68%, specificity 76%.
(2.21 MB TIF)

Methods S1 Diagnostic algorithms
(0.03 MB DOC)

Table S1 Head-to-head comparison of Quantiferin in tube test (QFT-IT), IP-10 test and IL-2 test
(0.03 MB DOC)

Table S2 Head-to-head comparison of Tuberculin Skin Test (10 mm cut off), IP-10 test and IL-2 test
(0.03 MB DOC)
