# Supplementary material for: Improving T-Cell Assays for the Diagnosis of Latent TB Infection: Potential of a Diagnostic Test Based on IP-10
Source: PLoS One. 2008 Aug 6;3(8):e2858. doi: 10.1371/journal.pone.0002858 (PMC2483344; doi:10.1371/journal.pone.0002858)
Supplement: Methods S1 — Diagnostic algorithms (0.03 MB DOC) [file pone.0002858.s002.doc]

**Methods S1:**

In a previous study antigen- and mitogen-dependent IP-10 responses from 80 patients with culture or PCR positive tuberculosis and 86 healthy controls were used to develop a IP-10 test algorithm with positive, negative and indeterminate test outcomes (Ruhwald et at, submitted). Cut-offs for a positive test was estimated using ROC-curve analysis on the antigen-dependent values. A range of potential cut-offs from 237pg/ml (sensitivity 90%, specificity 95%) to 673pg/ml (specificity 100%, sensitivity 74%) were identified. A cut off for positive test at 455pg/ml (sensitivity 80% specificity 97%) half way between these two extremes was pragmatically selected. A cut off for indeterminate test outcome was arbitrarily selected at 200pg/ml mitogen-dependent IP-10 production.

For IL-2 we used a predefined cut off from a small study on 11 healthy controls and 19 TB patients[17]. The cut off for positive test was set at mean antigen-dependent IL-2 concentration in the control group +3 standard deviations, at 13pg/ml. As the levels of antigen-induced IL-2 were low and close to the un-stimulated (nil) levels, we additionally set the criterion that in order for a response to be positive the antigen-dependent IL-2 response should be at least 125% of the un-stimulated value. As there was no mitogen-dependent IL-2 produced, it was not possible to include an option for indeterminate IL-2 test outcome.
