# Supplementary material for: Improving T-Cell Assays for the Diagnosis of Latent TB Infection: Potential of a Diagnostic Test Based on IP-10
Source: PLoS One. 2008 Aug 6;3(8):e2858. doi: 10.1371/journal.pone.0002858 (PMC2483344; doi:10.1371/journal.pone.0002858)
Supplement: Table S1 — Head-to-head comparison of Quantiferin in tube test (QFT-IT), IP-10 test and IL-2 test (0.03 MB DOC) [file pone.0002858.s003.doc]

**Table S1:**

| QFT-IT | IP-10 test | IL-2 test | N | % |
| --- | --- | --- | --- | --- |
|  |  |  |  |  |
| negative | negative | negative | 37 | 31 |
| negative | negative | positive | 1 | 1 |
| negative | positive | negative | 5 | 4 |
| negative | positive | positive | 2 | 2 |
| negative | indeterminate | negative | 7 | 6 |
| negative | indeterminate | positive | 1 | 1 |
| Positive | negative | positive | 3 | 3 |
| Positive | positive | positive | 45 | 38 |
| indeterminate | negative | negative | 5 | 4 |
| indeterminate | positive | negative | 1 | 1 |
| indeterminate | positive | positive | 3 | 3 |
| indeterminate | indeterminate | negative | 10 | 8 |
|  |  |  |  |  |
|  |  | ∑ | 120 | 100 |
