# Supplementary material for: Improving T-Cell Assays for the Diagnosis of Latent TB Infection: Potential of a Diagnostic Test Based on IP-10
Source: PLoS One. 2008 Aug 6;3(8):e2858. doi: 10.1371/journal.pone.0002858 (PMC2483344; doi:10.1371/journal.pone.0002858)
Supplement: Table S2 — Head-to-head comparison of Tuberculin Skin Test (10 mm cut off), IP-10 test and IL-2 test (0.03 MB DOC) [file pone.0002858.s004.doc]

**Table S2**

| TST | IP-10 test | IL-2 test | n | % |
| --- | --- | --- | --- | --- |
|  |  |  |  |  |
| Negative | negative | negative | 36 | 32 |
| Negative | negative | positive | 2 | 2 |
| Negative | positive | negative | 4 | 4 |
| negative | positive | positive | 15 | 14 |
| negative | indeterminate | negative | 16 | 14 |
| negative | indeterminate | positive | 1 | 1 |
| positive | negative | negative | 3 | 3 |
| positive | negative | positive | 2 | 2 |
| positive | positive | negative | 1 | 1 |
| positive | positive | positive | 30 | 27 |
| positive | indeterminate | negative | 1 | 1 |
|  |  |  |  |  |
|  |  | ∑ | 111 | 100 |
